# Factors affecting microbial safety behavior of beef handlers working in major beef retailers in Mizan-Aman, Southwest Ethiopia

**DOI:** 10.1371/journal.pone.0326862

**Published:** 2025-07-03

**Authors:** Girma Mamo Zegene, Seid Tiku Mereta, Seblework Mekonen

**Affiliations:** 1 Department of Public Health, Mizan Aman Health Science College, Mizan Aman, Ethiopia; 2 Department of Water and Health, Ethiopian Institute of Water Resources, Addis Ababa University, Addis Ababa, Ethiopia; 3 Departments of Environmental Health Science and Technology, Director, Center for One Health, Jimma University, Jimma, Ethiopia; University of Georgia, UNITED STATES OF AMERICA

## Abstract

**Background:**

Beef is a key component of human diet but also a favorable medium for microbial growth. However, research has largely overlooked major beef retailers and the specific roles of handlers, missing critical points for intervention. This study aims to address these gaps to support targeted microbial safety measures based on handlers’ specific roles in beef processing.

**Methods:**

A cross-sectional study was conducted in Mizan-Aman, Southwest Ethiopia, from February 20 to April 20, 2024. The sample size was determined using a single population proportion formula, resulting in the inclusion of 372 participants, yielding a response rate of 360 (96.8%). Beef retailers were randomly selected, while beef handlers were chosen through systematic sampling technique. Data collection was done using a structured questionnaire and observation checklist. Following a quality assessment, the data were analyzed using STATA 16, employing descriptive statistics and logistic regression. Bivariable analysis (p ≤ 0.25) was performed to identify variables for multivariable analysis. Statistical significance was assessed using adjusted odds ratios (AOR) and 95% confidence intervals (CI), with a significance level set at p < 0.05.

**Results:**

Findings indicated that a significant proportion of beef handlers demonstrated inadequate knowledge (61%), negative attitudes (58%), and insufficient safety practices (55%). Among several factors associated with knowledge, attitude and practice of beef handlers, training in food safety was linked to good knowledge (AOR = 4.17, 95% CI: 1.15–15.12), while the role of a waiter was associated with both good knowledge (AOR = 4.5, 95% CI: 1.83–10.95), and more favorable attitudes (AOR = 2.8, 95% CI: 1.02–7.31). On the other hand, poor knowledge (AOR = 4.40, 95% CI: 2.44–7.94) and unfavorable attitudes (AOR = 4.84, 95% CI: 2.62–8.95) were significantly correlated with inadequate microbial safety practices.

**Conclusion:**

Many beef handlers lack sufficient knowledge, attitudes, and practices regarding beef safety, regardless of their job roles. Improving microbial safety requires strategies such as formal education, training, health checks, and certification.

## 1. Introduction

Foodborne diseases (FBDs) remain a leading public health concern, with approximately 600 million (1 in 10 people) foodborne illnesses occurring each year [1], In developing countries, up to 2 million deaths are reported related with annually [2], These figures are often underreported and poorly documented due to insufficient surveillance systems in healthcare facilities and the tendency of some patients to seek treatment from traditional healers [3,4].

Despite its nutritional benefits, beef and beef products serve as suitable growth media for a variety of microorganisms [5]. Microbial contamination of beef can occur at any point along the farm-to-consumption chain [6]. An international study on foodborne outbreaks from 1996 to 2005 indicated that 12.7% of these outbreaks were linked to bovine meat [7]. In sub-Saharan African countries, bacteria are the dominant cause of foodborne outbreaks [8]. Notably, human salmonellosis and pathogenic strains of Escherichia coli (E. coli) causes to diarrhea, urinary tract infections, sepsis, meningitis, and pneumonia accounted for 32.9% and 34.6% respectively [7].

The perishable nature of meat underscores the importance of knowledge, health, and training among beef handlers to ensure consumer safety [9]. The microbial safety of beef is heavily influenced by the behavior of beef handlers and the infrastructures present at all levels of the beef supply chain [10]. Inadequate performance regarding microbial safety among beef handlers, together with insufficient infrastructure, plays a significant role in the transmission of beef-borne diseases [11,12]. Poor knowledge and inadequate safety practices among beef handlers adversely affect the microbial quality of meat [13], and their attitudes can further negatively influence their knowledge and practices [14].

In developed countries, beef handlers have shown increasing awareness of safety measures related to beef [15]. However, in developing countries, these problems persist, with insufficient studies identifying differences in knowledge, attitudes, and practices (KAP) within beef handlers’ duties and across major beef retailers [16,17].

Formal education, training, health examinations, and certification are key components in strategies aimed at improving microbial safety and the proficiency of beef handlers in compliance with safety requirements [12,18]. Education focused on behavioral change is essential for all roles in the beef supply chain, including waiters, cooks, and dish cleaners [19]. Additionally, possessing knowledge alone is not enough to foster positive attitudes and safe practices [20],but it must be coupled with effective perception and implementation of safety measures [21]. This study seeks to evaluate beef handler KAP status to design context-specific awareness and skill-building initiatives. Furthermore, the findings will fill the need to upgrade insights toward beef safety to properly meet an increasing demand for safe and processed beef driven by urbanization and lifestyle changes. The findings of the recent study will provide updated information for food safety officers, policymakers, and food establishment owners, helping them identify where appropriate actions should be taken.

## 2. Methods

### 2.1. Study design

A cross-sectional study design was carried out among beef handlers working in major beef retailers in the supply chain. Beef suppliers and retailers are deemed adequate for the study as the beef supply chain is monitored by the International Livestock Research Institute (ILRI) in Ethiopia [22]. This study aimed to assess beef handlers’ knowledge, attitudes, and practices (KAP) regarding microbial safety measures in terms of their duties across various types of beef retailers.

### 2.2. Study area and period

This study was conducted in Southwest Ethiopia Peoples Region (SWEPR), Bench Sheko Zone Mizan-Aman town. Bench Sheko Zone has six administrative districts, covering a total area of 19,252 km^2^ [23]. Bench Sheko Zone is a well-known place for animals’ products, vegetables, fruits, and root production in southwest Ethiopia. Particularly, beef and beef products are the favorite, cultural, and common food commodities among urban and rural residents. Mizan-Aman the capital town of Bench Sheko Zone, located at 6°57’25“ N latitude and 35°32’37” E longitude, with an elevation of 1275 m [24]. Recently, Mizan-Aman has been designated as one of the reform towns in the newly emerged region (SWEPR) with a city administration of third-grade status.

According to Mizan-Aman administration office, the population of residents is 121,755. Of this population, 50.3% are males and the rest 49.7% were females. In Mizan-Aman town, there are one health center, teaching hospital, university, vocational and technical school and health and agriculture colleges which are publicly owned. Furthermore, there are several privately owned clinics and drug dispensaries for human and animal health care.

A list of beef producers and retailers was obtained from Mizan-Aman town administration offices, revealing 2 municipally owned abattoirs, 29 butcher shops, 47 hotels, and 68 restaurants (including informal establishments). Due to significant fluctuations in beef handler numbers, a preliminary survey was conducted across all randomly chosen beef retail outlets during morning, afternoon, and weekend sessions. The study was carried out between February 20th and April 20th, 2024.

### 2.3. Sample size

The sample size was calculated using a single population proportion formula, assuming a 95% confidence interval and a 5% margin of error. A 32.6% population proportion of good microbial safety practices, based on a study in Arba Minch, Ethiopia [25], was used to ensure an adequate sample size as follows:


$$n=\frac{{\left(za/2\right)}^{2})*p(q)}{{\;d}^{2}}$$

where: n = required sample size, Z = Z-score (1.96 for 95% confidence), p = estimated proportion (0.326), q= (1-p), and d = margin of error (0.05)


$$n=\;\frac{\;{\left(1.96\right)}^{2}(0.326)(1-0.326)}{\;({0.05)}^{2}}\;n=\;338$$

Finally, the sample size including 10% non-response rate was 372.

### 2.4. Inclusion and exclusion criteria

This study included all beef retailers providing beef and beef products, regardless of their licensing status, and actively working beef handlers, irrespective of their socioeconomic differences. Retailers that had disrupted their beef product offerings and beef handlers unable to participate due to serious illness were excluded

### 2.5. Sampling procedures and technique

**Fig 1:**
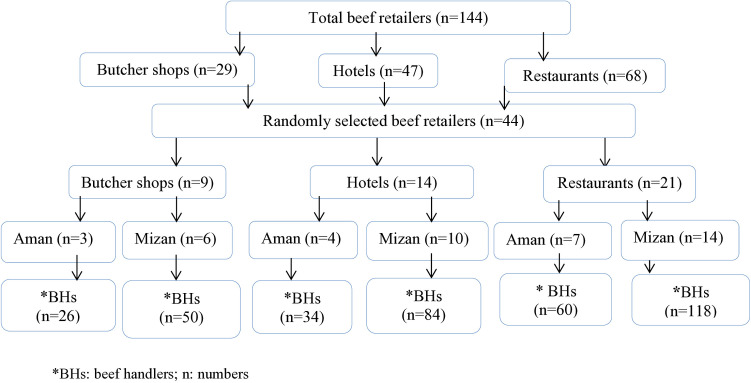
Beef retailers and study participants selection procedures.

Beef retailers and potential participants were proportionally allocated to Mizan and Aman towns based on their total numbers. Beef retail outlets (butcher shops, hotels, and restaurants) were then selected using a lottery method. To account for staffing variations, a preliminary survey registered all beef handlers (butchers, kitchen workers, waiters, and dish cleaners) in each selected outlet, resulting in a total of 1,488 (N = 1,488). A sample size of 372 participants was selected via systematic sampling (interval k = 4 [26,27], with proportional allocation of the n = 44 outlets based on handler numbers. Within each outlet, handlers were numbered sequentially, and a random number generator (range 1–4) determined the starting point for participant selection. Subsequent participants were selected every 4^th^ handler until the target sample size reached (Fig 1).

### 2.6. Data Collection

Data were collected by trained, and local-language-proficient data collectors, using a three-scale, close-ended questionnaire to gather information on participants’ sociodemographic (14 questions), knowledge (17 questions), attitudes (17 questions), and practices (17 questions). The questionnaire was administered face-to-face to systematically selected interviewees working in randomly selected retail outlets. Simultaneously, an observation checklist was used. After obtaining informed verbal consent [28], data were collected through the Kobo Collect mobile app and uploaded to the Kobo Toolbox server. Supervisors were managed the data collection process and gave feed backs directly.

### 2.7. Data quality management

To ensure data quality, the questionnaire and observation checklist, adapted from prior studies [18,29], and translated into the local language and back-translation into English. A pretest was conducted in neighboring Bonga town using 5% of the total sample size. Throughout the data collection period, supervisors provided continuous oversight and immediate feedback, and daily reviews of submitted data were performed. The data, initially imported from the Kobo Collect server into Excel, were then exported to STATA 16 for analysis. Data completeness and consistency were assessed, and a multicollinearity test yielded a mean Variance Inflation Factor (VIF) of 1.91. Furthermore, the Hosmer-Lemeshow test was conducted, resulting in a non-significant p-value of 0.26 [30].

### 2.8. Operational definitions

#### Beef handler.

An individual involved in the handling of beef products, including butchers (male or female), waiters, kitchen staff, and utensil cleaners in beef retail establishments [9,31].

#### Beef retailers.

Establishments such as butcher shops, hotels, and restaurants which prepare, process, distribute or sell beef and its products to customers or consumers

#### Butcher shops.

Retail outlets primarily focused on selling raw, semi-processed, and processed meat to customer

#### Hotels.

Establishments that offer accommodations including bedrooms with private baths, telephones, televisions, laundry services, coffee shops, dining rooms, cocktail lounges, and business conference halls, which may also feature associated butcher shops [32].

Poor knowledge: Beef handlers who score below the mean value of all knowledge questions was categorized as having poor knowledge [29,33,34].

#### Poor practice.

Beef handlers who score below the mean value of all practice questions was categorized as having poor practice [29,33,34].

#### Restaurants.

Commercial establishments that prepare and serve takeout food, beef products, and beverages to custom.

#### Unfavorable attitude.

Beef handlers who score below the mean value of all attitude questions toward beef safety was categorized as having unfavorable attitude [29,33,34].

### 2.9. Data processing and analysis

Data were analyzed using STATA 16.0. Mean scores were used to categorize beef handlers as having good or poor knowledge/practice and favorable or unfavorable attitudes [28,35]. Descriptive analysis and logistic regression were performed. Variables with p ≤ 0.25 in bivariable analysis were included in multivariable analysis [30,36,37]. Statistical significance was determined at p ≤ 0.05, with adjusted odds ratios (AOR) and 95% confidence intervals (CI) reported.

### 2.10. Ethical approval

Permission to conduct this research was granted by Jimma University institutional review board (IBR) with the approval letter Ref. No. JUIH/IRB/047/24 on February 14, 2024. Subsequently, we have obtained support letters from all concerned governmental and private organizations in Mizan-Aman, Southwest Ethiopia. All the research procedures were performed based on internationally acknowledged research ethics and principles. As per the guidelines, before commencing an interview and observations, all participants were informed about the aim and purpose of conducting this study by providing an approved information sheet and consent form. After all, data collection was preceded when an autonomous decisions and oral agreements obtained from participants.

## 3. Results

### 3.1. Socio-demographic characteristics

Of 372 beef handlers surveyed across 44 randomly selected retailers, 360 (96.8%) responded. The majority were female (55%) and young adults aged 21–25 (36.9%), with a mean age of 24.1 years (SD + 6.9). Most (88.6%) had not received safe beef handling training, and 81.94% had never undergone medical checkups (Table 1).

**Table 1:** Socio-demographic characteristics of beef handlers working in (n = 44) beef retailers.

Variable	Category	Frequency (%)
Address	Mizan	252(70.0)
	Aman	108 (30.0)
Sex	Male	162 (45.0)
	Female	198 (55.0)
Age in years	≤ 20	108 (30.0)
	21-25	133 (37.0)
	26-30	64 (17.8)
	≥ 31	55 (15.2)
Educational level	Not read and write	71 (19.7)
	Elementary school	186 (51.7)
	High school and above	103 (28.6)
Safe food handling training	Never trained	319 (88.6)
	Trained at least once	41 (11.4)
Medical check ups	Never checked	295(81.94)
	Checked at least once	65 (18.06)
Beef retailer type	Butcher shops	77 (21.4)
	Hotels	120 (33.3)
	Restaurants	163(45.3)
Participants’ duty	Butcher man/woman	48 (13.3)
	Waiter	190 (52.8)
	Kitchen worker	81 (22.5)
	Utensils cleaner	41 (11.4)
Salary (ETB/month)	≤ 2000	206 (57.2)
	2001-4000	136 (37.8)
	≥4001	18 (5.0)
Work experience (in years)	≤1	154 (42.8)
	2-5	191(53.0)
	6-9	15 (4.2)
FSIS	Radio	8 (2.2)
	Television	32 (8.9)
	Internet	6 (1.7)
	Co-worker	130 (36.1)
	Family	45 (12.5)
	School	62 (17.2)
	Health professionals	77 (21.4)
Knowledge	Good	141 (39.17)
	Poor	219 (60.83)
Attitude	Favorable	153(42.50)
	Unfavorable	207 (57.50)
Practice	Good	161 (44.72)
	Poor	199 (55.28)

**Abbreviation:** ETB: Ethiopian Birr; FSIS: food safety information source.

**Fig 2:**
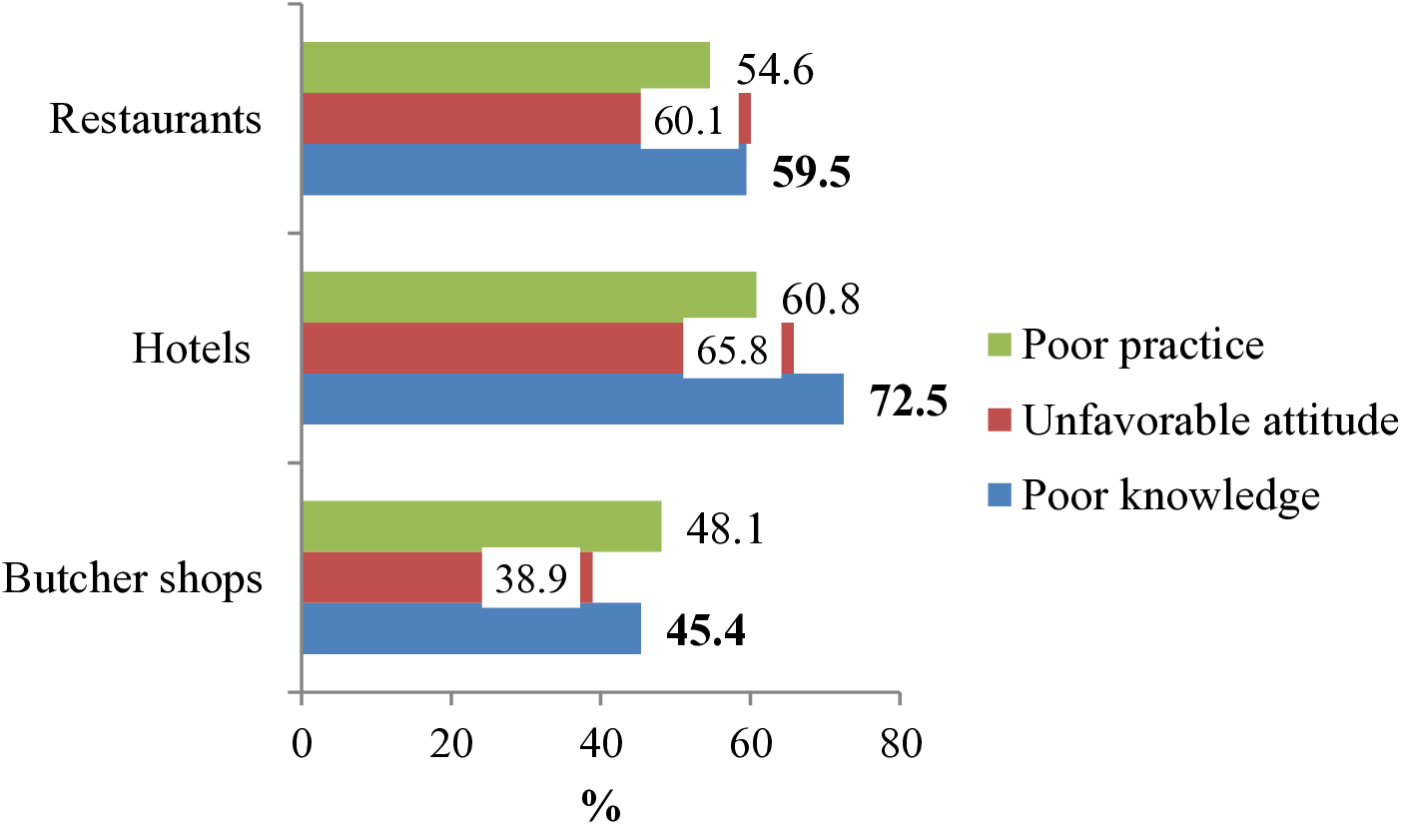
Beef handlers’ KAP level in major beef retailers.

Compared to butcher shop employees, beef handlers in hotels (65.8%) and restaurants (60.1%) had a more unfavorable attitude toward microbial safety measures. Furthermore, hotel employees showed a higher prevalence of poor microbial safety practices (60.8%) (Fig 2).

**Fig 3:**
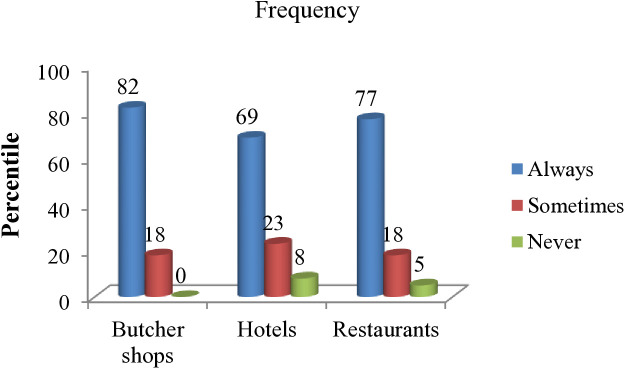
Availability and usage of functional refrigerators in beef retailers.

Approximately 82% of butcher men and women, 77% of restaurant staff, and 69% of hotel employees reported that they have functional refrigerators and regularly store beef products (Fig 3).

**Fig 4:**
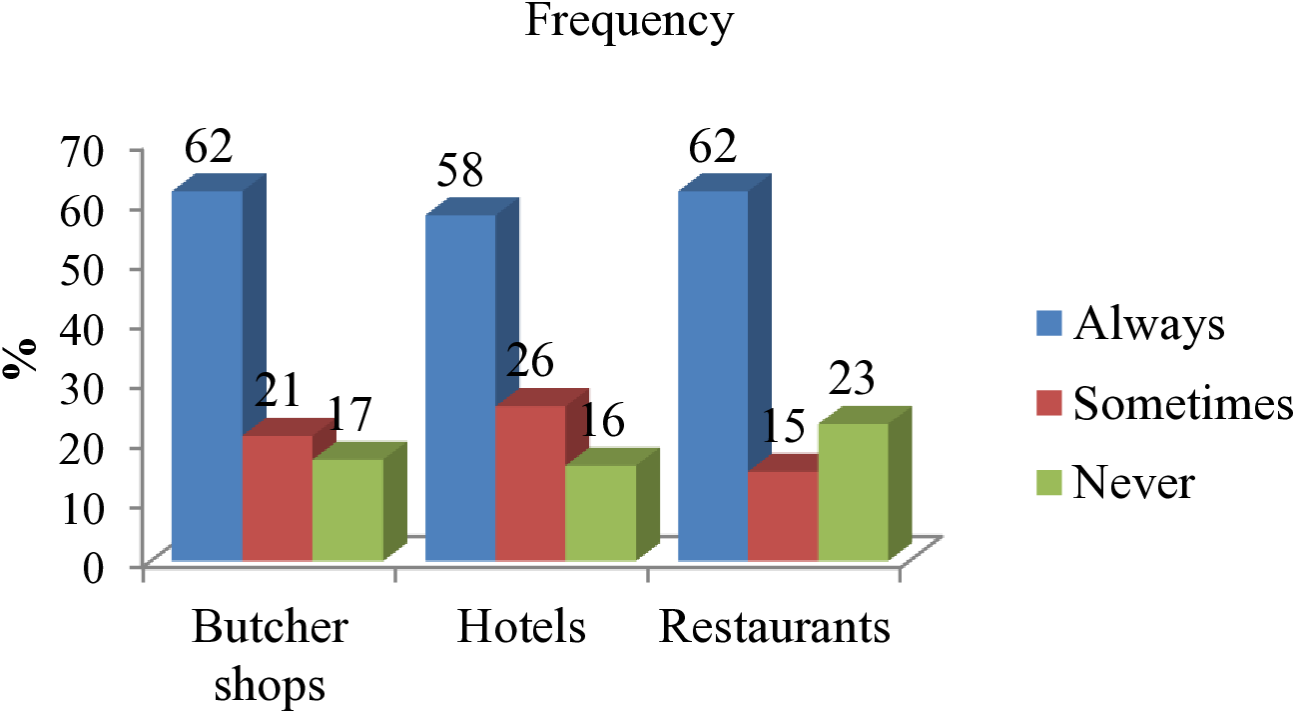
Practice of separating beef and organ by-products during storage.

However, a significant portion of employees in butcher shops (62%), restaurants (62%), and hotels (58%) reported that they often display and store beef products together with by-products or visceral organs (Fig 4).

**Fig 5:**
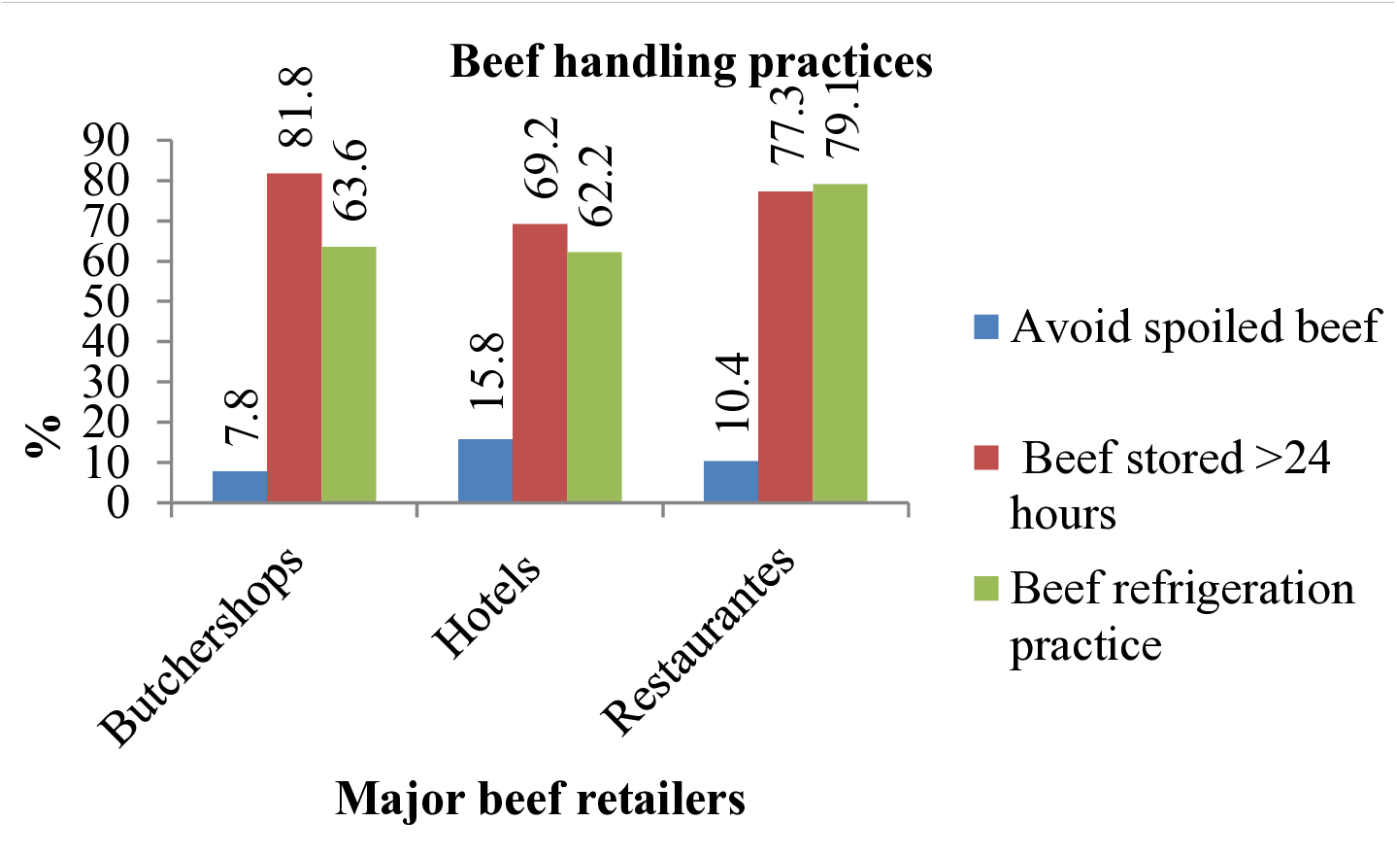
Beef handlers’ habits regarding microbial safety practices.

In butcher shops, 81.8% of beef handlers were stored processed beef for over a day frequently, while 0nly 7.8%. of participants replied that that they have been avoiding spoiled beef. Similarly, 69.2% of hotel employees and 77.3% of restaurant staff reported that they have tend of storing beef products longer than 24 hours (Fig 5).

**Fig 6:**
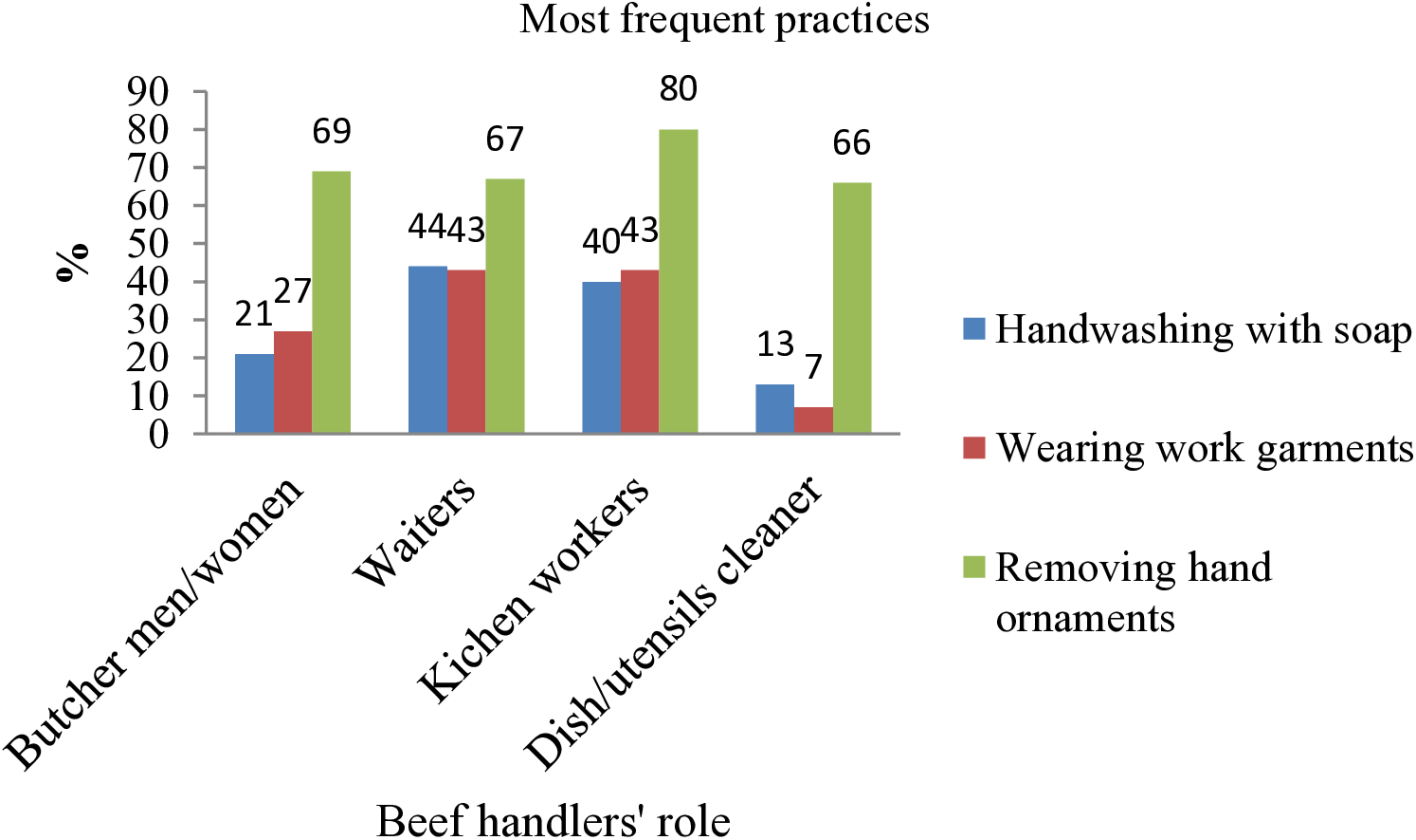
Microbial safety practices of beef handlers by job role.

The majority of butcher men or women (69%), waiters (67%), kitchen workers (80%), and utensil cleaners (66%) did not wear hand ornaments while on duty. The microbial safety practices of beef handlers by their job roles, a small proportion of butcher shop workers (men and women) (21%), waiters (44%), kitchen workers (40%), and utensil cleaners (13%) washed their hands with soap and safe water after every interruption during their duty (Fig 6).

**Fig 7:**
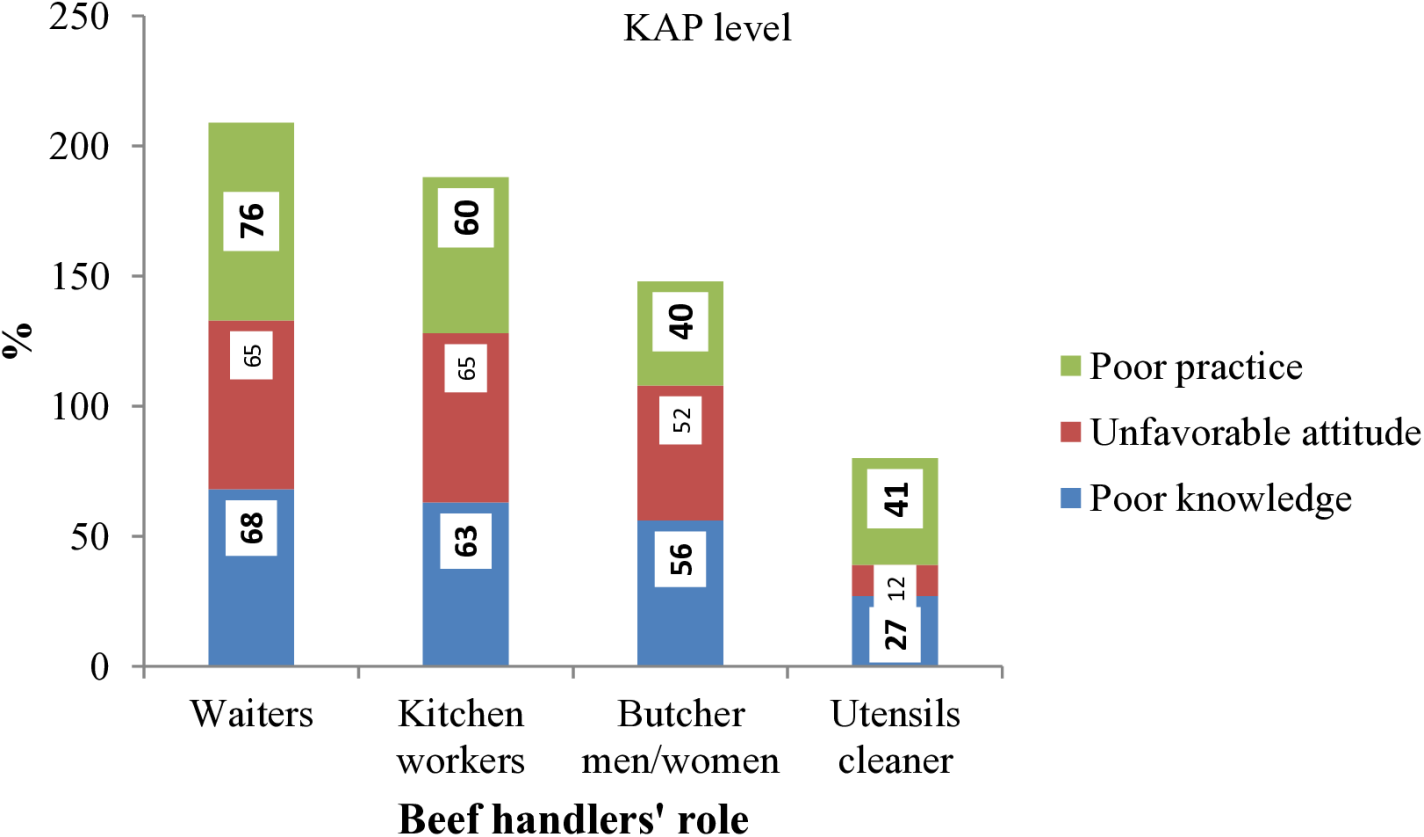
KAP levels of beef handlers by job roles.

Moreover, majority of waiters (76%), kitchen workers (60%), utensil cleaners (41%), and butchers (40%) exhibited poor microbial safety practices (Fig 7).

### 3.2. Factors associated with knowledge

Beef handlers aged over 31 years were four times more likely to demonstrate good knowledge of beef safety measures compared to those under 20 years of age (AOR = 3.74, 95% CI: 1.35–10.37, p = 0.011). Similarly, handlers trained in safe food handling were four times more likely to possess good knowledge compared to those who were never trained (AOR = 4.17, 95% CI: 1.148–15.123, p = 0.030).

Beef handlers working in hotels were four times more likely to show good knowledge compared to those in butcher shops (AOR = 4.01, 95% CI: 1.99–8.46, p = 0.001). Likewise, those working in restaurants were twice as likely to have good knowledge compared to their counterparts in butcher shops (AOR = 2.0, 95% CI: 1.037–3.873, p = 0.038).

Beef handlers earning 2001–4000, and ≥ 40,001 ETB monthly were twice and three times more likely to demonstrate good knowledge compared to those earning less than 2,000 ETB (AOR = 2.36, 95%CI:1.18–4.73,p = 0.016, AOR = 3.9, 95% CI: 2.89–29.90, p = 0.005) respectively (Table 2).

**Table 2:** Multivariable logistic regression analysis regarding knowledge of beef handlers toward microbial safety measures.

Variable	Category	Knowledge		AOR (95%CI)	p-value
		Good (%)	Poor (%)		
Age (years)	≤ 20	60 (55.6)	48 (44.4)	1	
	21-25	46 (34.6)	87 (65.4)	1.34[0.69–2.57]	0.379
	26-30	25 (39.1)	39 (60.9)	1.02[0.45–2.33]	0.961
	≥ 31	45 (81.8)	10 (18.2)	3.74[1.35–10.37]	*0.011
Education	Not read & write	43 (60.6)	28 (39.4)	1	
	Elementary school	78 (41.9)	108 (58.1)	1.18[0.61–2.29]	0.630
	High school and above	20 (19.4)	83 (80.6)	2.04[0.85–4.88]	0.111
FST	Never trained	138 (43.3)	181 (56.7)	1	
	Trained	38 (92.7)	3 (7.3)	4.17[1.15–15.12]	*0.030
Beef retailer	Butcher shops	42 (54.5)	35(45.5)	1	
	Hotels	87 (72.5)	33 (27.5)	4.10[1.99–8.46]	*0.001
	Restaurants	97 (59.5)	66 (40.5)	2.00[1.04–3.87]	*0.038
Beef handlers’ duty	Butcher man/woman	21(43.7)	27(56.3)	1	
	Waiter	130 (68.4)	60 (31.6)	4.48[1.83–10.95]	*0.001
	Kitchen worker	30 (37.0)	51 (63.0)	1.71[0.68–4.33]	0.252
	Utensils cleaner	30 (73.2)	11(26.8)	2.10[0.62–6.83]	0.238
Salary (ETB/month)	≤2000	100 (48.5)	106 (51.5)	1	
	2001-4000	96 (70.6)	40(29.4)	2.36[1.18–4.73]	* 0.016
	≥4001	17 (94.4)	1 (5.6)	3.9[2.89–29.90]	*0.005
Work experience(years)	≤1	83 (53.9)	71 (46.1)	1	
	2-5	55 (28.8)	136 (71.2)	1.53[0.87–2.69]	0.138
	≥6	3 (20.0)	12 (80.0)	1.25[0.26–5.97]	0.775
Medical checkup	Never	127 (43.1)	168 (56.9)	1	
	At least once	14 (21.5)	51 (78.5)	1.45[0.65–3.24]	0.363

**Notes:** *Significant at p ≤ 0.05.

**Abbreviations:** AOR: adjusted odds ratio; COR: crude odds ratio; FST: food safety training.

### 3.3 Factors associated with attitude

Beef handlers who completed high school and above were three times more likely to have a favorable attitude toward beef safety measures compared to individuals who could not read and write (AOR = 3.30, 95% CI: 1.30–8.35, p = 0.012). Beef handlers working in hotels were five times more likely to exhibit a favorable attitude toward beef safety measures compared to those working in butcher shops (AOR = 4.56, 95% CI: 2.12–9.81, p = 0.001). Conversely, beef handlers working in restaurants were three times less likely to hold a favorable attitude toward beef safety measures compared to those in butcher shops (AOR = 3.14, 95% CI: 1.52–6.48, p = 0.002).

In terms of duty, waiters were four times more likely to have a favorable attitude toward beef safety measures compared to butchers (AOR = 3.54, 95% CI: 1.40–8.92). Beef handlers who earned ≥4001 ETB monthly and had 2–5 years of working experience were twelve times more likely to demonstrate a favorable attitude toward microbial safety measures compared to those earning ≤2000 ETB per month (AOR = 11.7, 95% CI: 1.72–79.70, p = 0.012). Similarly, handlers with 2–5 years of experience were two times more likely to have a favorable attitude compared to those with ≤1 year of experience (AOR = 2.0, 95% CI: 1.15–3.52, p = 0.015) (Table 3).

**Table 3:** Multivariable logistic regression analysis regarding attitude of beef handlers toward microbial safety measures.

Variable	Category	Attitude		AOR (95%CI)	p-value
		Favorable (%)	Unfavorable (%)		
Sex	Male	76 (46.9)	86 (53.1)	1	
	Female	121 (61.1)	77(38.9)	1.0[0.58–1.83]	0.926
Age in years	≤ 20	69 (63.9)	39 (36.1)	1	0.494
	21-25	52 (39.1)	81 (60.9)	1.26[0.65–2.45]	0.316
	26-30	20 (31.3)	44 (68.7)	1.55[.66–3.63]	0.112
	≥ 31	12 (21.8)	43 (78.2)	2.23[0.83–6.01]	
Education	Not read and write	46 (64.8)	25 (35.2)	1	
	Elementary school	90 (48.4)	96 (51.6)	1.16[0.57–2.38]	0.686
	High school and above	86 (83.5)	17(16.5)	3.30[1.30–8.35]	*0.012
FST	Never trained	149 (46.8)	170 (53.2)	1	
	Trained at least once	4 (9.8)	37 (90.2)	2.86[0.90–9.13]	0.076
Beef retailer	Butcher shops	47 (61.0)	30 (39.0)	1	
	Hotels	79 (65.8)	41 (34.2)	4.56[2.12–9.81]	*0.001
	Restaurants	98 (60.1)	65 (39.9)	3.14[1.52–6.48]	*0.002
Beef handlers’ duty	Butcher man/woman	23 (47.9)	25 (52.1)	1	
	Waiter	124 (65.3)	66 (34.7)	3.54[1.40–8.92]	*0.007
	Kitchen worker	28 (34.6)	53 (65.4)	2.43[0.91–6.51]	0.077
	Utensils cleaner	36 (87.8)	5 (12.2)	0.86[0.22–3.42]	0.834
Salary (ETB/month)	≤2000	111 (53.9)	95 (46.1)	1	
	2001-4000	40 (29.4)	96 (70.6)	1.85[0.93–3.70]	0.082
	≥4001	16 (88.9)	2 (11.1)	11.7[1.72–7.79]	*0.012
Experience (in years)	≤1	97 (63.0)	57 (37.0)	1	
	2-5	136 (71.2)	55(28.8)	2.0[1.15–3.52]	*0.015
	6-9	1 (6.7)	14 (93.3)	8.62[0.93–79.61]	0.058
Medical checkup	Never	138 (46.8)	157 (53.2)	1	
	At least once	15 (23.1)	50 (76.9)	1.52[0.67–3.48]	0.318S

**Notes:** *Significant at p ≤ 0.05.

**Abbreviations:** AOR: adjusted odds ratio; COR: crude odds ratio; FST: food safety training.

### 3.4. Factors associated with practice

Beef handlers aged 26–30 were 33% more likely to perform good microbial safety practices compared to individuals under 20 years of age (AOR = 0.33, 95% CI: 0.13–0.82, p = 0.017). Similarly, beef handlers above 31 years of age were 25% more likely to exercise good microbial safety practices compared to individuals under 20 years of age (AOR = 0.25, 95% CI: 0.08–0.78, p = 0.017). In addition, beef handlers who received training in safe food handling were eight times more likely to apply good microbial safety practices compared to those who were never trained (AOR = 8.02, 95% CI: 1.70–37.77, p = 0.008).

Waiters and kitchen workers were four and three times less likely apply microbial safety practices compared to butcher shop workers [(AOR = 3.84, 95%CI: 1.37–10.80, p = 0.011, and AOR = 2.75, 95% CI: 1.02–7.41, p = 0.045)] respectively. Similarly, dish or utensil cleaners were five times less likely to accomplish microbial safety measures compared to butchery workers (AOR = 4.75, 95% CI: 1.33–16.94, p = 0.016). Beef handlers with a monthly salary of ≥4001 ETB and 2–5 years of working experience were significantly linked with the application of good microbial safety practices compared to those earning ≤ 2000 ETB per month (AOR = 7.15, 95% CI: 1.03–49.68, p = 0.047 and AOR = 1.93, 95% CI: 1.04–3.58, p = 0.037) respectively.

Participants who demonstrated poor knowledge and unfavorable attitudes toward beef safety measures were four and five times less likely to perform microbial safety practices compared to their counterparts with good knowledge and favorable attitudes regarding microbial safety measures (AOR = 4.04, 95% CI: 2.43–7.94, p = 0.001 and AOR = 4.84, 95% CI: 2.62–8.95, p = 0.001), respectively (Table 4).

**Table 4:** Multivariable logistic regression analysis regarding practice of beef handlers toward microbial safety measures.

Variable	Category	Practice		AOR (95%CI)	p-value
		Good (%)	Poor (%)		
Address	Mizan	106 (42.1)	146 (57.9)	1	
	Aman	55 (50.9)	53(49.1)	0.67[0.38–1.19]	0.175
Age in years	≤ 20	54 (50.0)	54 (50.0)	1	
	21-25	57 (42.9)	76 (57.1)	0.50[0.24–1.05]	0.066
	26-30	34 (53.1)	30 (46.9)	0.33[0.13–0.82]	*0.017
	≥ 31	35 (63.6)	20 (36.4)	0.25[0.08–0.78]	*0.016
Education	Not read &write	38 (53.5)	33 (64.5)	1	
	Elementary school	96 (51.6)	90 (48.4)	0.62[0.30–1.26]	0.186
	≥ High school	27 (26.2)	76 (73.8)	1.0[0.39–2.56]	0.997
FST	Never trained	159 (49.8)	160 (50.2)	1	
	Trained	39 (95.1)	2 (4.9)	8.02[1.70–37.77]	*0.008
Beef retailer	Butcher shops	40 (51.9)	37 (48.1)	1	
	Hotels	47 (39.2)	73 (60.8)	0.70[0.32–1.51]	0.362
	Restaurants	74 (45.4)	89 (54.6)	0.72[0.35–1.47]	0.365
Beef handlers’ duty	Butcher man/woman	29 (60.4)	19 (39.6)	1	
	Waiter	76 (40.0)	114 (60.0)	2.75[1.02–7.41]	* 0.045
	Kitchen worker	32 (39.5)	49 (60.5)	3.84[1.37–10.80]	*0.011
	Utensils cleaner	24 (58.5)	17 (41.5)	4.75[1.33–16.94]	*0.016
Salary (ETB/month)	≤ 2000	105 (51.0)	101 (49.0)	1	
	2001-4000	54 (39.7)	82 (60.3)	1.10[0.53–2.29]	0.795
	≥4001	16 (88.9)	2(11.1)	7.15[1.03–49.68]	* 0.047
Experience (years)	≤1	93 (60.4)	61 (39.6)	1	
	2-5	126 (65.9)	65 (34.1)	1.93[1.04–3.58]	* 0.037
	6-9	3 (20.0)	12 (80.0)	2.73[0.52–14.23]	0.234
Medical checkup	Never checked	142 (48.1)	153 (51.9)	1	
	Checked	19 (29.2)	46 (70.8)	0.95[0.41–2.20]	0.907
Knowledge	Good	100 (70.9)	41(29.1)	1	
	Poor	61(27.9)	158 (72.1)	4.40[2.43–7.94]	*0.001
Attitude	Favorable	107 (69.9)	46 (30.1)	1	
	Unfavorable	54 (26.1)	153 (73.9)	4.84[2.62–8.95]	*0.001

**Notes:** *Significant at p ≤ 0.05.

**Abbreviations:** AOR: adjusted odds ratio; COR: crude odds ratio; FST: food safety training.

## 4. Discussion

### 4.1. Beef handlers’ KAP

This study revealed that more than half of beef handlers (BHs) demonstrated poor knowledge, unfavorable attitudes, and poor practices toward beef safety measures, aligning with findings in other regions in Ethiopia [38], yet contradictory to findings from studies conducted abroad in Ethiopia [9]. These differences may be attributed to variations in the implementation of food safety guidelines, regulations governing food catering practices, and food safety enforcement laws concerning employers and employees [18].

The mean scores of participants’ knowledge, attitudes, and practices (KAP) toward beef safety measures were 21.5 (±3.0), 21.2 (±3.5), and 24.0 (±4.9), respectively. These scores were significantly lower than those reported in a study from Khulna City, Bangladesh [21]. Employees of butcher shops showed a higher prevalence of inadequate microbial safety practices, which was lower than findings reported in Gondar, Ethiopia [39]. The major contributing factor for this difference might be the poor attention given to food handling training and formal education as a pre-requisite in this context [9]. A significant portion of participants (< 50%) did not adhere to microbial safety protocols by failing to wash their hands with soap after contact with soiled objects, their own body parts, or following interruptions during their duties [40]. Similarly, the majority of BHs did not undergo medical checkups and did not receive any form of food safety training before or after recruitment. A considerable number of participants judged contaminated beef based on changes in color, odor, and taste. As a result, they did not consider beef as a favorable growth medium for heterogeneous microorganisms. These misconceptions and violations of microbial safety measures might be due to lack of safety standards, including providing adequate hygiene and sanitation facilities, infrastructure, safe food handling training, and regular medical checkups for their employees [41–43].

Around half of beef handlers working in butcher shops, hotels, and restaurants reported storing beef and visceral organs separately. The majority of participants stored beef in functional refrigerators, assuming this would reduce microbial growth. In contrast, a study in Vietnam found that only 9% of food handlers in large canteens did not separate raw meat from cooked [19]. These results were consistent with a study from Bahir Dar, Ethiopia [44], but contrary to findings from studies in Western Romania [9], Turkish food businesses [45], Saudi Arabian restaurants [45], and in Nigeria [46]. The possible differences might be due to a lack of adherence to microbial safety standards, insufficient awareness creation [18], and continuous education and training [47].

### 4.2. Factors associated with beef handlers’ KAP

Beef handlers aged over 31 years showed significantly better knowledge of beef safety measures than those under 20, a finding contrary to previous Malaysian research [20]. This difference is likely due to greater experience and exposure to food safety practices [20]. Several factors were associated with better beef safety knowledge and more favorable attitudes among beef handlers. Specifically, those with safe food handling training, higher education (college or high school), higher monthly incomes, and experience working in hotels or restaurants, and waiter roles demonstrated good knowledge and favorable attitudes toward beef safety. This aligns with previous research suggesting that education and training possibly contributing in improving knowledge and attitude of beef safety activities in Jordanian [48], and with a study conducted in Lahore district, Pakistan [35]. Higher income may be a correlated factor, linked to both education and experience and attitude is also a fundamental factor influencing food safety practices [49].

Adult beef handlers aged 26–30 and ≥ 31 years, those trained in safe food handling, with 2–5 years of working experience, and earning ≥ 4001 ETB monthly incomes were more likely to apply good microbial safety practices compared to their counterparts. Higher income may be associated with better educational status, experience, and knowledge of food handling practices [34]. On the other hand, waiters, kitchen workers and utensil cleaners were less likely to realize microbial safety practices compared to butcher shop workers. Beef handlers with poor knowledge and unfavorable attitudes toward beef safety measures were poorly perform microbial safety practices compared to those participants with good knowledge and favorable attitude. This finding was aligned with studies conducted in Dangila town, Northwest Ethiopia [34], and Hawassa City, Southern Ethiopia [27], indicating a positive relationship between knowledge, attitude, and practice toward microbial safety measures.

Improved food safety practices are potentially linked to adequate knowledge, supported by on-site supervision and potentially higher salaries [9,19,27].

## 5. Conclusion

This study revealed significant deficiencies in beef safety knowledge, attitudes, and practices (KAP) among beef handlers, irrespective of their roles or working beef retailer types. Better outcomes were associated with education, training, experience, and higher wages, but a significant number of participants lacked these. Comprehensive and urgent interventions, focusing on training programs and rigorous adherence to microbial safety protocols, are needed. Beef retailers must ensure staff training and periodic health checks. Collaboration among regulatory bodies, industry, educational institutions, and the media is essential to enhance beef safety.

### Limitation

The current study did not evaluate the direct impact of beef handlers’ knowledge, attitudes, and practices (KAP) on the prevalence and load of microorganisms in beef products throughout the supply chain. Future research will aim to address this limitation particularly focusing on the prevention strategies for beef-borne diseases.
