# Correction: Factors affecting microbial safety behavior of beef handlers working in major beef retailers in Mizan-Aman, Southwest Ethiopia

**DOI:** 10.1371/journal.pone.0335553

**Published:** 2025-10-27

**Authors:** Girma Mamo Zegene, Seid Tiku Mereta, Seblework Mekonen

There are errors in the author affiliations. The correct affiliations are as follows:

Girma Mamo Zegene^1,3^, Seid Tiku Mereta^1^, Seblework Mekonen^2^

**1** Department of Environmental Health Sciences & Technology, Public Health Facult, Institute of Health Sciences, Jimma University, Jimma, Ethiopia, **2** Department of Water and Health, Ethiopian Institute of Water Resources, Addis Ababa University, Addis Ababa, Ethiopia, **3** Department of Public Health, Mizan Aman Health Science College, Mizan Aman, Ethiopia.
